# Axial Hysteretic Mechanical Characteristics of Wire Rope Isolators and Parameter Identification with a Novel Algebraic Closed-Form Model

**DOI:** 10.3390/ma19071452

**Published:** 2026-04-05

**Authors:** Gangwei Mei, Yongsheng He, Mengnan Dai, Longyun Zhou, Xiongliang Yao, Jun Shen, Chunhai Li

**Affiliations:** 1 College of Shipbuilding Engineering, Harbin Engineering University, Harbin 150001, China; 2 State Key Laboratory of Target Vulnerability Assessment, Beijing 100036, China; 3 Institute of Defense Engineering, AMS, PLA, Beijing 100036, China

**Keywords:** wire rope isolators, hysteresis curve, asymmetric hysteresis model, model parameter identification, axial loading test

## Abstract

Wire rope isolators (WRIs) exhibit typical nonlinear and asymmetric hysteretic behavior, with their mechanical performance being significantly influenced by the coupled effects of multiple parameters. This study investigates the dynamic response of large-sized spiral WRIs under axial loading. Within the framework of an asymmetric hysteresis model, a novel algebraic closed-form formulation is adopted for parameter identification and numerical simulation. Furthermore, a characteristic parameter, *A*, is introduced to quantify the unique mechanical behavior induced by the structural configuration of WRIs. Five types of large-sized spiral WRIs are selected as test specimens. For each WRI, tests are conducted under 30 distinct working conditions, yielding a total of 150 cyclic loading tests across all scenarios. By systematically varying the displacement amplitude, loading frequency, and preloading pressure, the influences of these key parameters on the dynamic characteristics of WRIs are comprehensively analyzed. These characteristics encompass the axial hysteresis loop shape, energy dissipation capacity, equivalent viscous damping, and average secant stiffness. The results indicate that these three loading parameters exert substantial effects on the mechanical properties of large-sized WRIs. Additionally, the simulated hysteresis curves derived from the identified parameters exhibit excellent agreement with the experimental observations. Compared with conventional mechanical models, the proposed algebraic closed-form model demonstrates slightly higher fitting accuracy, thereby validating its effectiveness and applicability in characterizing the mechanical behavior of large-sized WRIs. This research provides a crucial reference for the engineering application of large-sized spiral WRIs and facilitates the broader adoption of the proposed modeling approach.

## 1. Introduction

Impact and vibration control technologies play a pivotal role in modern engineering, particularly in high-end sectors such as aerospace, precision manufacturing, military equipment, and nuclear power facilities, where they have a direct bearing on equipment reliability, operational accuracy, and safety. Among the diverse range of vibration isolation devices, wire rope isolators (WRIs) distinguish themselves through their unique all-metal construction and intrinsic damping mechanisms, offering exceptional environmental adaptability and long-term stability. Consequently, WRIs serve an irreplaceable function in vibration and shock mitigation under extreme operating conditions [1,2,3,4]. The fundamental operating principle of WRIs derives from their ingenious geometric configuration: multiple strands of stainless steel wire are helically wound and clamped between upper and lower metallic end plates, forming an isolation structure that combines flexibility with pronounced damping capacity [5,6]. Under dynamic loading, mechanisms such as dry friction, localized inter-strand slippage, and macroscopic deformation—both between wire strands and between strands and end plates—act in synergy to establish an efficient energy dissipation system. This design affords WRIs outstanding isolation performance along all three orthogonal directions, enabling effective absorption and dissipation of vibrational energy across a wide frequency range. Their performance is markedly superior to that of many traditional rubber or hydraulic isolators. However, WRI performance is not dictated by a single factor but is instead the result of a complex interplay of multiple parameters. Effective stiffness, damping properties, and energy dissipation capacity are strongly influenced by design variables such as the isolator’s macro-geometry, wire strand diameter and count, number of winding turns, winding angle, and the magnitude and direction of applied loads [7,8,9,10]. This pronounced parameter sensitivity renders accurate mechanical modeling and reliable performance prediction highly challenging tasks. Comprehensive research into WRI mechanical modeling can not only deepen our understanding of their internal mechanical mechanisms but also provide a scientific basis for optimizing their performance in current applications and fostering innovative uses in emerging domains, including vibration isolation for high-precision instrumentation [11,12,13,14].

The mechanical behavior of WRIs is inherently nonlinear [15,16,17,18], with a key characteristic being the nonlinear hysteresis observed in the force–displacement relationship. Under large displacement amplitudes, this hysteresis often exhibits pronounced asymmetry—namely, disparities between the stiffness and energy dissipation characteristics along loading and unloading paths. Such asymmetric hysteresis loops are not unique to WRIs but are also observed in materials such as shape memory alloys NiTi, polymers, and composite materials [19,20,21]. Consequently, developing mathematical models capable of accurately representing such nonlinear phenomena has become a prominent research focus in mechanics and engineering. Among the various hysteresis modeling approaches, the Bouc–Wen model and its extended derivatives have gained widespread favor for their ability to describe hysteresis smoothly and continuously, and they are extensively applied in numerous engineering contexts [22,23,24]. To tackle asymmetry, researchers have proposed numerous modifications to the classical Bouc–Wen framework. For example, Karabutov et al. [25] developed an adaptive parameter identification method to enhance model convergence. Song et al. [26] proposed a parameter-fitting strategy for the generalized Bouc–Wen model using genetic algorithms, leveraging the integral relationships across six distinct stages of an asymmetric hysteresis loop-derived from the model’s differential equations to construct a performance function and introducing conditions that ensure the closure of smooth hysteresis loops, thereby achieving accurate fitting for markedly asymmetric experimental curves.

Nevertheless, despite their high accuracy, Bouc–Wen-type models face inherent limitations linked to their differential formulation: (1) low computational efficiency, as time-history analysis typically involves iterative solutions to first-order differential equations, leading to considerable computational costs; and (2) complex parameter identification, with parameters often lacking tangible physical interpretation and relying on sophisticated optimization algorithms prone to local convergence issues. To overcome these shortcomings, recent research has shifted toward closed-form hysteresis models based on algebraic relationships. In this regard, the studies conducted by Vaiana et al. [27] represent a milestone. They systematically introduced and developed a family of algebraic uniaxial phenomenological hysteresis models. In contrast to the Bouc–Wen model, these algebraic formulations eliminate the need for numerically solving differential equations, substantially reducing computational effort. In subsequent work [28,29], they established general formulas for such models and captured rate-independent hysteresis by solving scalar equations in generalized force space. Their formulations, particularly the exponential variant, can effectively replicate complex hysteresis behaviors that exhibit motion hardening or softening. Comparative analyses against the Bouc–Wen model confirmed the advantages of the algebraic approach in terms of both computational efficiency and accuracy. Despite the considerable progress achieved by researchers worldwide in characterizing and modeling the dynamic properties of WRIs, notable gaps remain—especially regarding large-scale WRIs subjected to large displacement amplitudes. Most existing studies concentrate on small-scale WRIs and nonlinear behavior within limited displacement ranges, leaving insufficient exploration of dynamic performance under large displacement conditions, particularly in terms of energy dissipation mechanisms and stiffness nonlinearities. While the novel algebraic closed-form model proposed by Vaiana [28] holds great promise, its applicability and robustness for large-scale WRIs operating under large displacements, varying excitation frequencies, and multi-parameter influences require extensive experimental validation.

In response to these issues, the present study undertakes systematic experimental research and theoretical analysis to investigate the nonlinear dynamic characteristics of WRIs under axial loading. Specifically, it examines the influence of critical factors—including displacement amplitude, excitation frequency, axial preload, and wire rope diameter—on the energy dissipation capacity and stiffness nonlinearity of large-scale WRIs, as well as model parameter identification under diverse operating conditions. A comprehensive experimental protocol was implemented, comprising 200 cyclic loading tests conducted in a laboratory environment. The resulting data were processed to determine dissipated energy, equivalent viscous damping coefficients, and tensile and compressive secant stiffness values, thereby quantifying WRI performance across different scenarios. Building on this dataset, a novel algebraic closed-form hysteresis model was employed to fit and validate the experimental results. Model parameters for each condition were successfully identified, and systematic comparisons between model predictions and experimental hysteresis loops were performed to assess the accuracy and efficacy of the proposed approach in representing the complex nonlinear response of large-scale WRIs subjected to large displacements, varying frequencies, and multi-parameter influences.

## 2. Experimental Setup and Operating Conditions

**Figure 1:**
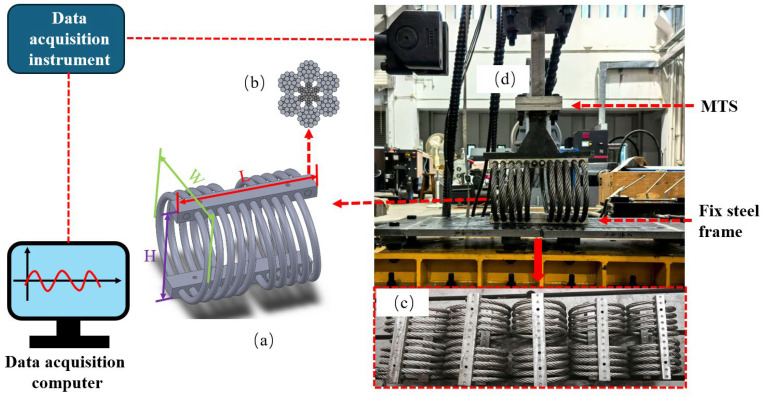
Schematic diagram of the test system, (**a**) wire rope isolator, (**b**) cross-section of the steel wire rope, (**c**) different types of wire rope isolator, (**d**) MTS loading rod.

The WRIs used in this experiment feature upper and lower endplates made from 316 stainless steel, with a steel wire rope helically wound between them, as illustrated in Figure 1a. The cross-sectional structure of the steel wire rope consists of seven strands of fine steel wires, with six strands wound around the central core of the primary strand, as shown in Figure 1b. The sliding friction between the strands and wires provides effective damping, which significantly reduces shock responses and absorbs subsequent vibrations.

Tests were performed on the wire rope isolator (WRIs) provided by Huanyu Shock Absorption Co., Ltd., Changzhou, China. The experiments were carried out on large-scale WRIs comprising five different models. The geometric characteristics of these wire rope isolators are summarized in Table 1 and illustrated in Figure 1c. These characteristics include the length *L*, height *H*, width *W*, aspect ratio *H*/*W*, wire rope diameter *D*, and structural parameter *A*, which is employed to evaluate the relative stiffness ranking of the isolators.

Testing was performed using the MTS793 fatigue testing machine, as shown in Figure 1. Force and displacement sensors were installed on the axial rod of the device. This equipment is capable of applying a maximum sinusoidal force of 500 kN and a maximum dynamic displacement of 300 mm. The WRIs were connected to the testing machine via a connecting rod.

A total of 40 axial tests were conducted on each WRI. The displacement amplitude was gradually increased from smaller to larger values to simulate the operating conditions of the isolators under varying vibration intensities. The tested displacement amplitudes were 5 mm, 10 mm, 20 mm, 30 mm, 40 mm, and 50 mm. Axial loading was applied through the testing system following a predefined loading protocol to replicate actual service conditions, with preset axial preloads of 0 kN, 1 kN, and 3 kN. The excitation frequencies were set to 0.2 Hz, 0.5 Hz, and 1 Hz.

For each combination of displacement amplitude |*A_d_*|, frequency *f*, and preload *P_N_*, eight sinusoidal displacement cycles were applied. Prior to testing, the rated load capacity of the testing equipment was verified, and the maximum allowable displacement and axial force were configured to prevent damage to the system. The detailed testing conditions are summarized in Table 2.

To elucidate the underlying control mechanism of the relative performance ranking of the five wire rope isolators (WRI), this paper proposes a geometric stiffness index (GSI), denoted as *A*. This index is a dimensionless indicator used to quantify the comprehensive impact of key geometric parameters such as height H, width B, and rope diameter d on the mechanical stiffness of the isolator.

Based on the simplified Beam–Column Theory and the force transmission mechanism inside the WRI structure, the stiffness *K* of the isolator is mainly determined by the geometric ratio. The stiffness *K* is proportional to the cross-sectional area (related to *d*^2^ and *B*) and inversely proportional to the effective force-bearing length (related to *H*). Based on this relationship, the definition of the geometric stiffness index A is derived as follows:

(1) $$A=\frac{H}{B}\times d$$

Here, *A* includes two key connotations: (1) the parameter d represents the inherent stiffness contribution of the core load-bearing wire rope itself, which is directly related to the cross-sectional area of the wire rope; and (2) the parameter *H*/*B* characterizes the aspect ratio of the structure, reflecting the stability and load-bearing efficiency of the overall geometric shape of the WRI. A larger *A* value corresponds to a combination of a larger rope diameter and an aspect ratio closer to 1 (i.e., more “square”), which geometrically indicates a higher potential stiffness. This provides a solid physical justification for using *A* as a descriptor for stiffness ranking in this study.

## 3. Analysis of Test Results

The geometric characteristics of the five WRI models are shown in Table 1, and each WRI underwent 40 axial tests. Each test yielded 8 valid force–displacement asymmetric hysteresis curves. From each test, 6 valid hysteresis curves were selected because the first and last curves may fail to close and contain errors due to structural instability. These two data points were discarded. Based on each test, the following four characteristic parameters of the axial dynamic properties of WRIs were investigated: (1) energy dissipation *E_d_*, (2) equivalent viscous damping coefficient *v_e_*, (3) maximum secant stiffness $k_{s}^{+}$, and (4) minimum secant stiffness $k_{s}^{-}$.

For each test, WRI force–displacement curves were plotted on a 2D plane, with tensile forces in the first quadrant and compressive forces in the third quadrant. This defined the maximum and minimum secant stiffness.

(2) $$k_{s}^{+}=\frac{\max(f)}{\max(d)}=\frac{f_{\max}}{d_{\max}},\;\mathrm{and}\;k_{s}^{-}=\frac{\min(f)}{\min(d)}=\frac{f_{\min}}{d_{\min}}$$

For symmetric hysteresis curves, the equivalent viscous damping coefficient $v_{e}$ is given by Equation (3):

(3) $$v_{e}=\frac{1}{4\pi }\frac{E_{d}}{E_{ev}}$$

(4) $$A_{ev}=\frac{1}{2}k_{s}d^{2}$$

For asymmetric hysteresis curves, Equation (3) needs modification due to differing secant stiffness in the tensile and compressive states. Kumar et al. [30] proposed a method to estimate the equivalent viscous damping factor by modifying the denominator of Equation (3), leading to Equation (4).

(5) $$v_{e}=\frac{1}{\pi }\frac{E_{d}}{A_{ev}^{I}+A_{ev}^{II}+A_{ev}^{III}}$$

The energies of, $A_{ev}^{I}$, $A_{ev}^{II}$ and $A_{ev}^{III}$ are calculated as follows:

(6) $$A_{ev}^{I}=\frac{1}{2}k_{s}^{+}d_{\max}^{2},\;A_{ev}^{II}=f_{\min}d_{\max},\;A_{ev}^{III}=\frac{1}{2}k_{s}^{-}d_{\min}^{2}$$

Each test records the force–displacement curve as a set of *n* samples [27,31], represented by the values *f_i_* − *d_i_* collected in the vector $\rho_{i}=(d_{i},f_{i})$. Accordingly, the dissipated energy associated $E_{d,j}$ with each hysteresis curve, denoted as, where *j* = 1, 2, 3, 4, 5, 6, is evaluated using Equation (7), that is:

(7) $$E_{d,j}=\frac{1}{2}\sum_{i=1}^{n}\rho_{i}\cdot \rho_{i+1}^{⊥}$$

The $\rho_{i+1}^{⊥}=(-f_{i+1},d_{i+1})[f_{i+1},d_{i+1}]$ value needs to be determined based on whether the hysteresis curve follows a clockwise or counterclockwise direction. By calculating the four parametric characteristics for the six hysteresis curves, the average of these four parametric characteristics is taken from the six hysteresis curves.

**Figure 2:**
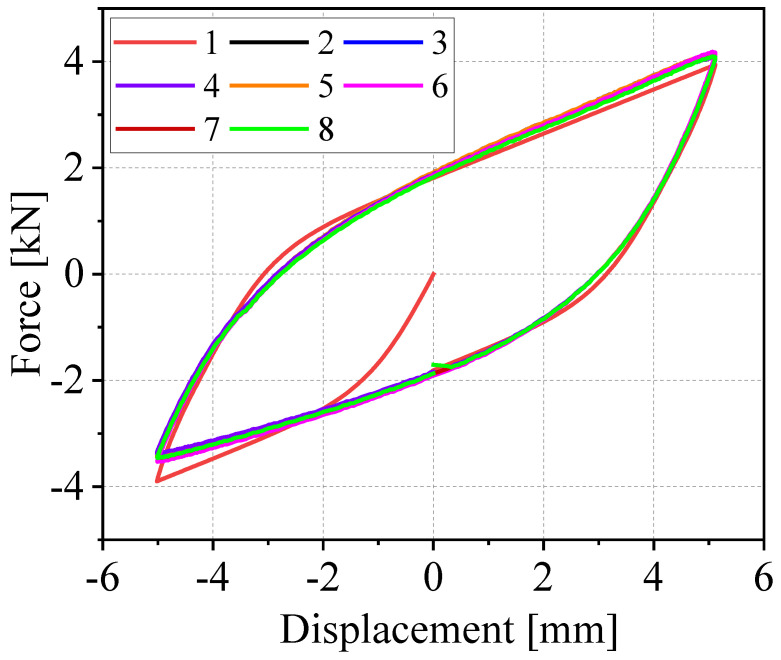
Eight hysteresis loops of the G300 vibration isolator at an amplitude of 5 mm.

As shown in Figure 2, taking the test results of the G300 vibration isolator under the condition of 0.2 Hz frequency and 5 mm amplitude as an example, the 8 groups of hysteresis loops exhibit distinct stage characteristics. Specifically, loops 2 to 7 demonstrate excellent closure and highly consistent morphologies, indicating that the vibration isolator has entered a stable cyclic loading state. By contrast, loops 1 and 8 are significantly unclosed and thus ineffective for characterizing the steady-state mechanical behavior of the specimen.

The non-closure of loop 1 is attributed to the initial transient effect of the test system. At the initial loading stage, the actuator exhibits a response delay, with hydraulic compression and mechanical transmission clearances not fully eliminated. Meanwhile, the interface gaps between the vibration isolator and the fixture are filled, leading to the force–displacement path being dominated by the assembly state. Consequently, synchronous force-displacement reset cannot be achieved during the unloading phase, resulting in the non-closure of the loop.

The non-closure of loop 8, on the other hand, is caused by the mechanical cumulative effect in the late cyclic loading stage. After multiple cyclic loadings, mechanical relaxation and slight residual deformation occur in the actuator, fixture, and the connection end of the specimen. Combined with the accumulation of hydraulic damping and friction, the unloading path deviates from the closed trajectory. Furthermore, the stress relaxation of the specimen further aggravates the offset at the end of the trajectory, leading to the non-closure of the loop. Therefore, only the stable hysteresis loops 2 to 7 are selected for subsequent analysis.

### 3.1. Analysis of Geometric Parameters and Stiffness Ranking

The geometric parameters of the five WRI types and the proposed geometric stiffness index *A* are summarized in Table 1. It can be seen from the data in the table that the order of *A* values clearly follows the stiffness ranking:

(8) $$A_{G240}<A_{G220}<A_{G280}<A_{G300}<A_{G280S}$$

**Figure 3:**
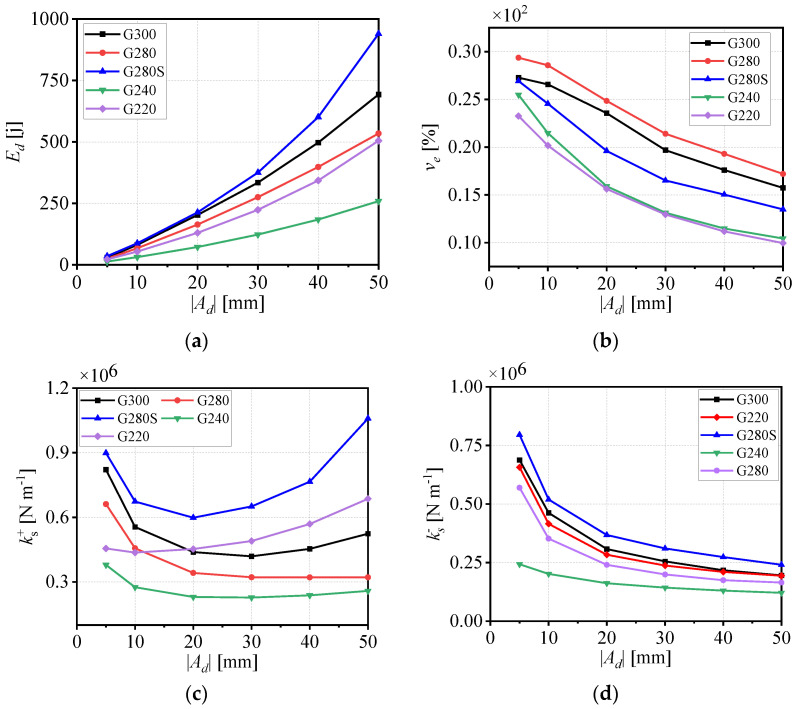
(**a**) Average dissipated energy *E_d_*, (**b**) average equivalent viscous damping factor *v_e_*, (**c**) average secant stiffness $k_{s}^{+}$, (**d**) average secant stiffness $k_{s}^{-}$ at 0.2 Hz for WRIs.

To explicitly verify the predictive ability of *A*, Figure 3 shows the correlation plots between various performance indicators (stiffness, dissipated energy, preload sensitivity) and the index *A*. The specific analysis is as follows:

(1) Stiffness Performance: As shown in Figure 3d, the vertical stiffness presents a strong positive correlation with *A*. This result confirms that *A* can effectively predict the stiffness ranking of isolators, among which Type V with the highest *A* value exhibits the largest stiffness, consistent with the theoretical derivation.
(2) Dissipated Energy: Figure 3a shows that the dissipated energy increases with an increase in *A*, indicating that a higher geometric stiffness index corresponds to better energy dissipation capacity, which further verifies the rationality of *A*.
(3) Analysis of Limitations: While *A* demonstrates strong predictive power for the primary rankings of stiffness and energy dissipation, it fails to fully capture complex nonlinear behaviors such as preload sensitivity—a property closely tied to friction and contact mechanics. This limitation will be addressed in subsequent research.
(4) In summary, the above analysis confirms that *A* is an effective predictive indicator for the main ranking of stiffness and energy dissipation, and also clarifies its limitations in secondary performance indicators.

### 3.2. Influence of Displacement Amplitude on Mechanical Properties

To investigate the effect of displacement amplitude |*A_d_*| on the performance of large-sized WRIs without axial pre-pressure, a loading frequency of 0.2 Hz was applied and the displacement amplitude |*A_d_*| was increased, as shown in Figure 2. Based on the hysteresis curves of the five isolators, four key parameters were examined, including the average dissipated energy *E_d_*, the average viscous damping coefficient *v_e_*, and the average secant stiffness values in tension $k_{s}^{+}$ and compression $k_{s}^{-}$, which were calculated using the relevant formulas. The dissipated energy *E_d_* is a crucial indicator of the energy dissipation capacity of WRIs, reflecting the amount of energy consumed by the isolator through damping during vibration. As shown in Figure 3a, *E_d_* increases significantly with the rise in displacement amplitude |*A_d_*| for all models. This is because, with increasing displacement amplitude, the relative sliding and friction between the internal steel wire contact surfaces become more pronounced, leading to greater energy dissipation. Among the different models, G280S exhibits the highest *E_d_* value and the fastest growth rate, followed by G300, while G280 shows a lower *E_d_* value than G300. The Ed value of G220 is similar to that of G300, while G240 demonstrates the lowest *E_d_* value. These differences are attributed to the structural parameters of the WRIs, such as the number of strands, twist pitch, and wire diameter. Larger values of parameter A lead to higher *E_d_* values under the same loading conditions. The viscous damping coefficient *v_e_* characterizes the damping properties of the steel wire isolators and reflects the relationship between energy dissipation and vibration parameters. As shown in Figure 3b, for isolators with the same steel wire diameter, the order of *v_e_* inversely correlates with the values of parameter *A*, i.e., G280 has a larger *v_e_* value than G300, that of G300 is larger than that of G280S, and that of G240 is larger than that of G220. For all models, the *v_e_* value decreases monotonically with increasing displacement amplitude |*A_d_*|. This is because, at smaller displacement amplitudes, the isolator primarily dissipates energy through viscous damping. As the displacement amplitude increases, the contribution of dry friction damping becomes more significant, reducing the relative contribution of viscous damping, which results in a decrease in the *v_e_* value.

The average secant stiffness values in tension $k_{s}^{+}$ and compression $k_{s}^{-}$ are important parameters that describe the stiffness characteristics of WRIs and directly affect the natural frequency and vibration isolation performance of the system. As shown in Figure 3c, the tensile stiffness $k_{s}^{+}$ of the different models shows varying trends as the displacement amplitude |*A_d_*| increases. For G300 and G280S, the $k_{s}^{+}$ value initially decreases and then increases. For G280 and G240, it first decreases and then stabilizes. G220 exhibits a generally increasing trend in $k_{s}^{+}$. G280S consistently maintains the highest $k_{s}^{+}$ value, indicating its superior ability to sustain load and maintain stiffness in tension. By contrast, G240 has the lowest $k_{s}^{+}$ value, reflecting the influence of wire diameter on stiffness. As shown in Figure 3d, the compressive secant stiffness $k_{s}^{-}$ value of all models decreases with increasing displacement amplitude |*A_d_*|. G280S has the highest initial $k_{s}^{-}$ value, followed by G300, G220, and G280. G240 consistently maintains a lower $k_{s}^{-}$ value. This indicates that, under compression, the stiffness of the wire rope isolator (WRI) is more sensitive to the wire diameter, height, and width of the wire rope isolator (WRI), meaning that the value of structural parameter A can effectively reflect this impact.

**Figure 4:**
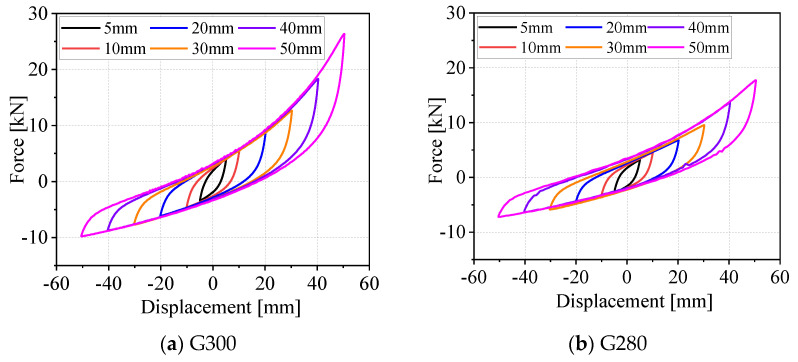

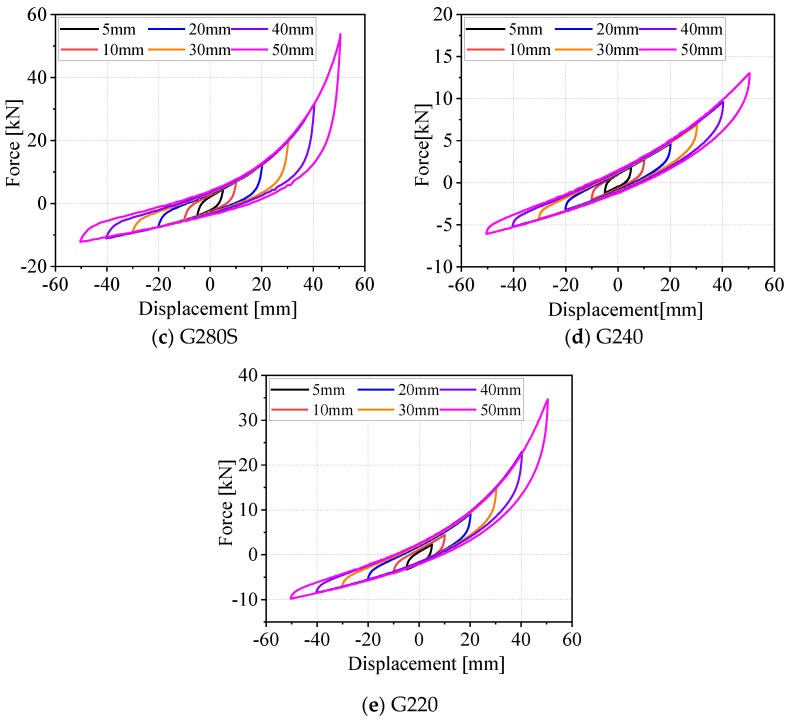
Dynamic hysteresis curves of different types of WRIs.

The hysteresis curves of the WRIs presented in Figure 4 clearly illustrate the energy dissipation and mechanical response characteristics of each isolator type. At a displacement amplitude of 50 mm, G280S exhibits the highest tensile and compressive forces, with a steeper curve and the largest hysteresis loop area, indicating its superior energy dissipation capacity. The curves for different displacement amplitudes are distinctly separated, demonstrating the significant impact of displacement amplitude on the mechanical performance of the isolators. For smaller rope diameters, the tensile and compressive forces of G240 are notably lower than those of the other models. Within the displacement range of −50 mm to 50 mm, the maximum bearing capacity third-quadrant force remains below 7 kN, and the overall hysteresis loop appears “slender,” suggesting relatively lower stiffness and energy dissipation capacity. By contrast, the hysteresis curve of G220 reaches a bearing capacity of nearly 10 kN at large displacement amplitudes 50 mm, highlighting its enhanced load-bearing and energy dissipation performance compared to smaller configurations.

### 3.3. Influence of Axial Preload on Mechanical Properties

**Figure 5:**
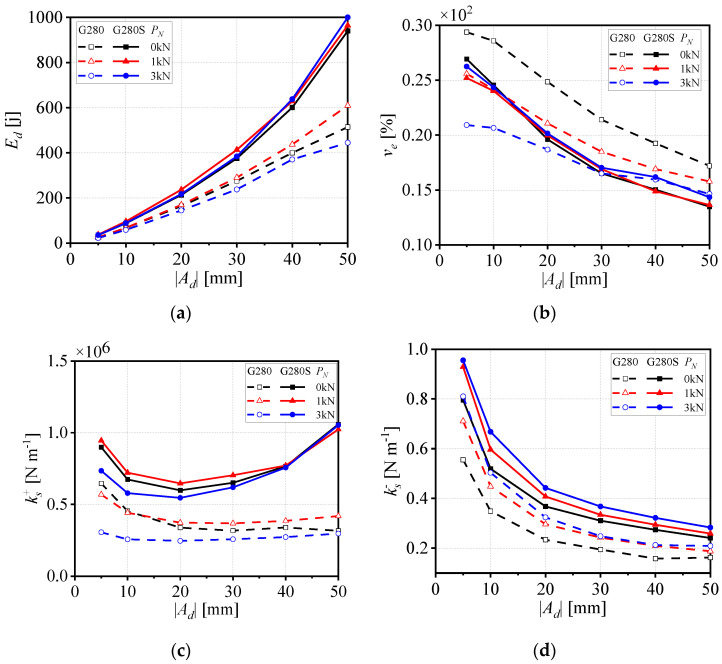
(**a**) Average dissipated energy *E_d_*, (**b**) average equivalent viscous damping factor *v_e_*, (**c**) average secant stiffness $k_{s}^{+}$, (**d**) average secant stiffness $k_{s}^{-}$.

The dynamic performance of WRIs is significantly influenced by the loading conditions, with axial preload pressure being a key factor. This pressure directly affects the internal contact interfaces within the WRIs, thereby altering their energy dissipation, damping, and stiffness characteristics. This section provides an in-depth analysis of the variations in energy dissipation *E_d_*, viscous damping coefficient *v_e_*, and average secant stiffness $k_{s}^{\pm }$ of G280 and G280S, which have the same height and rope diameter but differ in width, under axial pressures of 0 kN, 1 kN, and 3 kN. As shown in Figure 5a, for the G280 model, the *E_d_* value increases with pressure, with the order from smallest to largest being 3 kN, 0 kN, and 1 kN. For G280S, the *E_d_* values at different displacement amplitudes are relatively consistent. This is because, when the pressure increases to 1 kN, the internal contact pressure within the wire rope increases, which enhances the coupling effect between the friction coefficient and the friction area, leading to a significant increase in frictional energy dissipation. However, when the pressure reaches 3 kN, it exceeds the yield stiffness point of the WRIs, resulting in a reduction in the *E_d_* value during compression. Figure 5b shows that as the pressure increases, the *v_e_* value gradually decreases. The *v_e_* values for G280 vary significantly across different pressures, while the values for G280S exhibit relatively small differences. The tensile stiffness $k_{s}^{+}$ of G280S, as shown in Figure 5c, first decreases and then increases with increasing displacement amplitude at different axial pressures. By contrast, that of the G280 model initially decreases and then levels off, tending towards a horizontal trend. For both models, lower pressure results in lower initial stiffness. Under all pressure conditions, the $k_{s}^{-}$ value of G280S is consistently higher than that of G280, as shown in Figure 5d.

**Figure 6:**
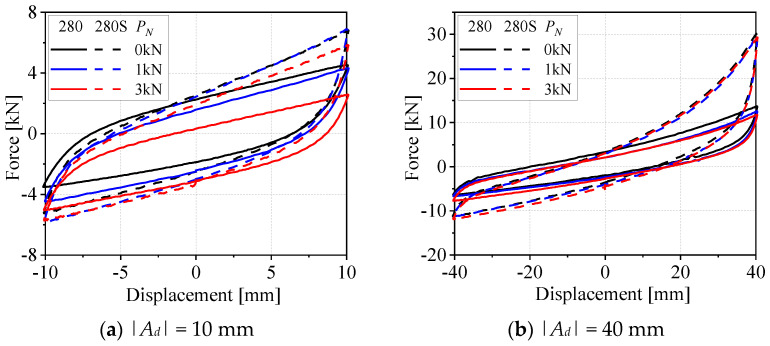
Hysteresis curves of two types of WRIs under different displacement amplitudes and preloads.

Figure 6 presents the force–displacement hysteresis curves of two wire rope isolator models (G280 and G280s) under different loading conditions, as shown in Figure 6a,b. Under axial preload application, the hysteretic response characteristics of the isolators vary significantly with displacement amplitude: at small displacement amplitudes, increasing the preload causes the maximum tensile force *f_max_* of both isolators to exhibit a decreasing trend, while their maximum compressive force *f_min_* shows an increasing trend. This is because the preload accounts for a relatively large proportion of the isolator stroke at small displacements. At this stage, the isolators operate in the pre-yield regime of their nonlinear mechanical response, where the influence of preload becomes more pronounced. As a result, the variation in the hysteresis loop area is negligible during this stage, as illustrated in Figure 6a. At large displacement amplitudes, isolators with a larger structural parameter A exhibit weaker preload dependence in their hysteresis curves, with curve profiles tending to be consistent across different preload levels. Conversely, for isolators with a smaller structural parameter *A*, the hysteresis loop area under preload application is significantly smaller than that under preload-free conditions (as shown in Figure 6b). The underlying reason for this mechanical behavior is as follows: isolators with a larger structural parameter *A* possess a higher overall stiffness, resulting in a relatively weak preload effect on their hysteresis curves; by contrast, isolators with a smaller structural parameter *A* have a lower overall stiffness, and thus the preload exerts a more pronounced influence on their hysteresis curves.

### 3.4. Influence of Frequency on Mechanical Properties

As a fundamental parameter of vibration systems, frequency plays a crucial role in regulating the energy dissipation, damping, and stiffness characteristics of WRIs. This section focuses on two WRI models, G280 and G280S, to investigate the variations in energy dissipation *E_d_*, viscous damping coefficient *v_e_*, and average secant stiffness $k_{s}^{\pm }$ under different excitation frequencies 0.2 Hz, 0.5 Hz, and1 Hz. The purpose is to provide theoretical support for the frequency-domain adaptability design of vibration isolation systems.

**Figure 7:**
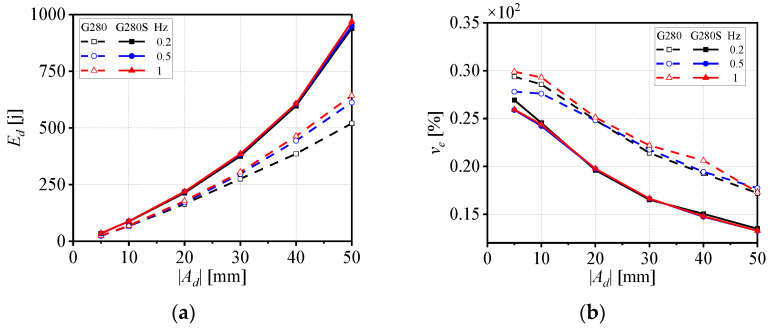

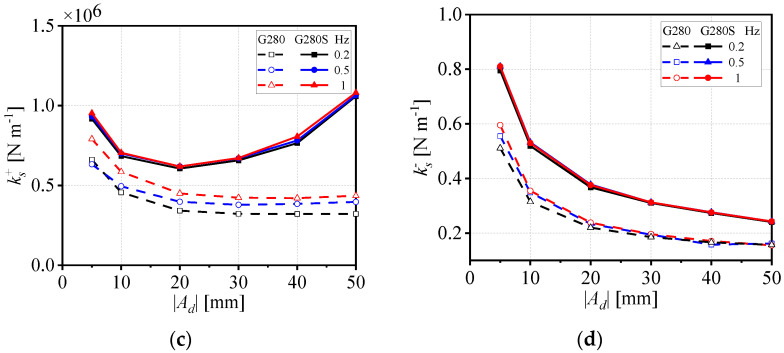
(**a**) Dissipated energy *E_d_*, (**b**) viscous damping coefficient *v_e_*, and average secant stiffness (**c**) $k_{s}^{+}$ and (**d**) $k_{s}^{-}$ of two types of WRIs under different frequencies.

As illustrated in Figure 7a, taking G280 as an example, the *E_d_* value increases progressively with frequency. Although the frequency has a relatively minor effect on *E_d_* in G280S, both models exhibit the same overall trend. This is because, as frequency increases, the number of frictional contact cycles within the wire rope per unit time rises, resulting in a stronger “cumulative effect” of frictional energy dissipation and, consequently, a significant increase in the *E_d_* value. Figure 7b indicates that frequency has little influence on the viscous damping coefficient *v_e_* for both models. From Figure 7c,d, it can be observed that under varying frequencies, the tensile stiffness $k_{s}^{+}$ of G280S and G280 changes differently with displacement amplitude |*A_d_*|. The G280S model exhibits higher $k_{s}^{+}$ values than G280 at all frequencies and shows a trend of initially decreasing and then increasing with larger |*A_d_*| value. For the G280 model, the $k_{s}^{+}$ value decreases initially and then tends to stabilize as the |*A_d_*| value increases, and the overall stiffness increases slightly with higher frequency. The compressive stiffness $k_{s}^{-}$ of both models decreases as the |*A_d_*| value increases, and frequency has only a minor effect on $k_{s}^{-}$. Moreover, G280S consistently exhibits higher $k_{s}^{-}$ values than G280 at different frequencies.

**Figure 8:**
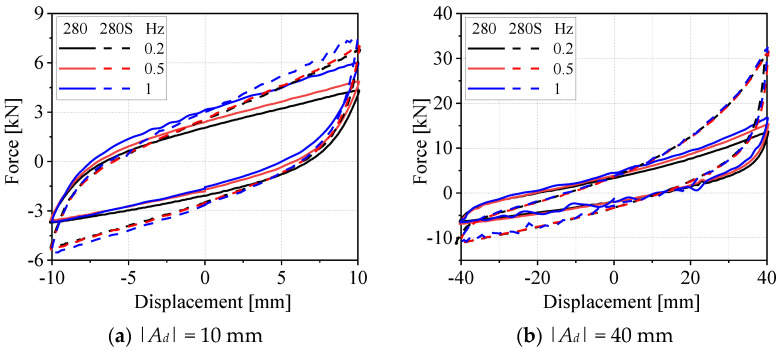
Hysteresis curves of two types of WRIs under different displacements and frequencies.

Figure 8 presents the force–displacement hysteresis curves of G280 and G280s steel wire rope isolators under various excitation frequencies and displacement working conditions. Evidently, the mechanical response characteristics reflected by the curves reveal that as the excitation frequency increases from 0.2 Hz to 1 Hz, the hysteresis loop areas of the two isolator types both exhibit a pronounced increasing tendency. Furthermore, as the excitation frequency rises, the maximum tensile force of the isolators in the tension phase shows a marked increase, whereas the maximum compressive force in the compression phase also increases yet with a considerably milder magnitude of growth. This distinct disparity in the variation magnitudes of the maximum tensile and compressive forces stems inherently from the structural features specific to steel wire rope isolators: their mechanical response to excitation frequency is considerably more sensitive in the tensile state, whereas this sensitivity diminishes markedly in the compressive state once the stress exceeds the yield point. As a key quantitative indicator for characterizing the energy dissipation capacity of isolators under dynamic loading conditions, the increase in hysteresis loop area is a direct reflection of the enhanced energy dissipation efficiency of the isolators. Combined with the aforementioned force response characteristics, it can be concluded that the output force of the G280 and G280s steel wire rope isolators exhibits an overall increasing tendency with the rise in excitation frequency. Within the medium-low frequency range of 0.2–1 Hz, both the energy dissipation capacity and dynamic load force of the two isolator types demonstrate favorable response sensitivity to excitation frequency.

## 4. Novel Algebraic Closure Model and Its Parameter Identification

### 4.1. Novel Algebraic Hysteretic Mechanical Model

**Figure 9:**
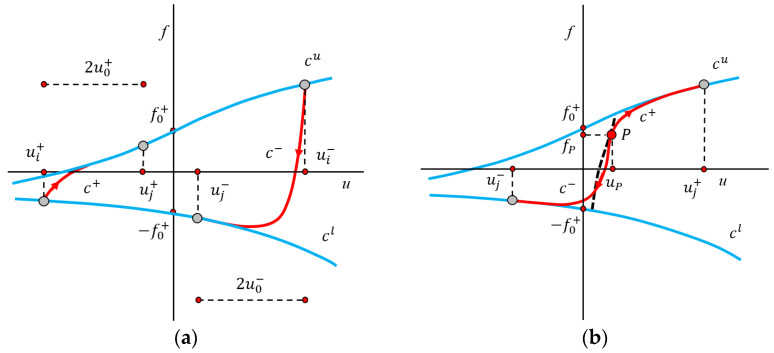
Curves *c*^+^, *c*^−^, *c*_*u*_, *c*_*l*_, (**a**) and internal variables $u_{j}^{+}$ and $u_{j}^{-}$ (**b**) for a generic asymmetric hysteresis loop.

The experimental results in Section 3 show that wire rope isolators (WRIs) exhibit nonlinear mechanical behavior under axial loading, and their force–displacement relationship under such loading can be characterized by an asymmetric hysteresis loop enclosed by two convex curves without inflection points. Based on the research findings of Vaiana et al. [28,29] on the novel algebraic closed-form model, this section carries out the parameter identification work for the model. This model overcomes the limitations of the improved Bouc–Wen model by: (1) calculating output variables in a closed form, eliminating the need for numerical solutions of differential equations; and (2) using two distinct sets of parameters to simulate the loading and unloading curves characterized by different tangent stiffness values. It is assumed that the general hysteresis curve is divided into four different curves: the general loading and unloading curves, denoted as *c*^+^ and *c*^−^ respectively, the upper and lower limit curves, denoted as $c_{u}$ and $c_{l}$, respectively. The force and displacement are represented by the symbols *f* and *d*, respectively. This section only presents the main derivation process. For the detailed derivation process, please refer to References [28,29], as shown in Figure 9.

#### 4.1.1. General Loading Curve $c^{+}$

When condition $u=u_{j}^{+}$ is satisfied, under the boundary condition that $c^{+}$ intersects $c_{u}$ at $u_{j}^{+}$, the general loading curve $c^{+}$ is equal to the upper limit curve $c_{u}$:

(9) $${\bar{f}}_{e}^{+}(u_{j}^{+})+f_{h}^{+}(u_{j}^{+},u_{j}^{+})={\bar{f}}_{e}^{+}(u_{j}^{+})+k_{b}^{+}u_{j}^{+}+f_{0}^{+}$$

Substitute the hysteretic force $f_{h}^{+}(u_{j}^{+},u_{j}^{+})$ into the formula (when $u=u_{j}$ is satisfied, the exponential term is $e^{-\alpha^{+}(u_{j}^{+}-u_{j}^{+}+{\bar{u}}^{+})}=e^{-\alpha^{+}{\bar{u}}^{+}}$):

(10) $${\bar{f}}_{e}^{+}(u_{j}^{+})+k_{b}^{+}u_{j}^{+}-\frac{1}{\alpha^{+}}e^{-\alpha^{+}(u-u_{j}^{+}+{\bar{u}}^{+})}+C_{h1}^{+}+C_{h2}^{+}={\bar{f}}_{e}^{+}(u_{j}^{+})+k_{b}^{+}u_{j}^{+}+f_{0}^{+}$$

After simplification, the integral constant of the hysteretic force is obtained as follows:

(11) $$C_{h1}^{+}+C_{h2}^{+}=f_{0}^{+}+\frac{1}{\alpha^{+}}e^{-\alpha^{+}{\bar{u}}^{+}}$$

Combining the elastic force formula and the hysteretic force formula, the final result is obtained as follows:

(12) $$\begin{array}{l} c^{+}(u,u_{j}^{+})=\beta_{1}^{+}(e^{\beta_{2}^{+}u}-1)+2\gamma_{1}^{+}\left(\frac{2}{1+e^{-\gamma_{2}^{+}(u-\gamma_{3}^{+})}}-1\right)+k_{b}^{+}u+\\ f_{0}^{+}-\frac{1}{\alpha^{+}}[e^{-\alpha^{+}(u-u_{j}^{+}+{\bar{u}}^{+})}-e^{-\alpha^{+}{\bar{u}}^{+}}] \end{array}$$

#### 4.1.2. Upper Limit Curve $c_{u}$

When condition $u>u_{j}^{+}$ is satisfied, $k_{h}^{+}=k_{b}^{+}$ holds; after integration, the result is obtained as follows:

(13) $$c_{u}(u)=\beta_{1}^{+}(e^{\beta_{2}^{+}u}-1)+2\gamma_{1}^{+}\left(\frac{2}{1+e^{-\gamma_{2}^{+}(u-\gamma_{3}^{+})}}-1\right)+k_{b}^{+}u+f_{0}^{+}$$

#### 4.1.3. Unloading Phase Curve $c^{-}$

It is only necessary to replace the parameters of the loading phase with those of the unloading phase (such as $k_{b}^{+}\to k_{b}^{-},f_{0}^{+}\to -f_{0}^{-},\alpha^{+}\to \alpha^{-}$), and the derivation process is completely symmetric. The final result is obtained as follows:

(14) $$\begin{array}{l} c^{-}(u,u_{j}^{-})=\beta_{1}^{-}(e^{\beta_{2}^{-}u}-1)+2\gamma_{1}^{-}\left(\frac{2}{1+e^{-\gamma_{2}^{-}(u-\gamma_{3}^{-})}}-1\right)+k_{b}^{-}u\\ -f_{0}^{-}+\frac{1}{\alpha^{-}}[e^{-\alpha^{-}(-u+u_{j}^{-}+{\bar{u}}^{-})}-e^{-\alpha^{-}{\bar{u}}^{-}}] \end{array}$$

#### 4.1.4. Lower Limit Curve $c_{l}$


(15) $$c_{l}(u)=\beta_{1}^{-}(e^{\beta_{2}^{-}u}-1)+2\gamma_{1}^{-}\left(\frac{2}{1+e^{-\gamma_{2}^{-}(u-\gamma_{3}^{-})}}-1\right)+k_{b}^{-}u-f_{0}^{-}$$

#### 4.1.5. Internal Variable $u_{j}^{\pm }$

$u_{j}^{+}$ is the coordinate of the intersection point of $c^{+}$ and $c_{u}$, which needs to satisfy the condition that “$c^{+}$ passes through any point $P(u_{P},f_{P})$”. Substituting $u=u_{P}$ and $f=f_{P}$:

(16) $$u_{j}^{+}=u_{P}+{\bar{u}}^{+}+\frac{1}{\alpha^{+}}ln\left(+\alpha^{+}[{\bar{f}}_{e}^{+}(u_{P})+k_{b}^{+}u_{P}+f_{0}^{+}+\frac{1}{\alpha^{+}}e^{-\alpha^{+}{\bar{u}}^{+}}-f_{P}]\right)$$

Similarly, $u_{j}^{-}$ can be obtained:

(17) $$u_{j}^{-}=u_{P}-{\bar{u}}^{-}-\frac{1}{\alpha^{-}}ln\left(-\alpha^{-}[{\bar{f}}_{e}^{-}(u_{P})+k_{b}^{-}u_{P}-f_{0}^{-}-\frac{1}{\alpha^{-}}e^{-\alpha^{-}{\bar{u}}^{-}}-f_{P}]\right)$$

#### 4.1.6. Derivation of Internal Parameter $u_{0}^{\pm }$

$u_{0}^{+}$ is 1/2 of the displacement difference between the start and end points of the loading curve, which needs to satisfy the condition that “$c^{+}$ intersects $c_{l}$ at $u=u_{j}^{+}-2u_{0}^{+}$”. Substituting $u=u_{j}^{+}-2u_{0}^{+}$ into the equality of $c^{+}$ and $c_{l}$:

(18) $$\begin{array}{l} \beta_{1}^{+}e^{\beta_{2}^{+}(u_{j}^{+}-2u_{0}^{+})}-\beta_{1}^{-}e^{\beta_{2}^{-}(u_{j}^{+}-2u_{0}^{+})}-\beta_{1}^{+}+\beta_{1}^{-}+2[\gamma_{1}^{+}\left(\frac{2}{1+e^{-\gamma_{2}^{+}(u_{j}^{+}-2u_{0}^{+}-\gamma_{3}^{+})}}-1\right)\\ -2\gamma_{1}^{-}(\left(\frac{2}{1+e^{-\gamma_{2}^{-}(u_{j}^{+}-2u_{0}^{+}-\gamma_{3}^{-})}}-1\right))]+(k_{b}^{+}-k_{b}^{-})(u_{j}^{+}-2u_{0}^{+})+\frac{\delta_{k}^{+}}{\alpha^{+}}(1-e^{2\alpha^{+}u_{0}^{+}})\\ +f_{0}^{+}+f_{0}^{-}=0 \end{array}$$

Similarly, $u_{0}^{-}$ is 1/2 of the displacement difference between the start and end points of the unloading curve, which needs to satisfy the condition that “$c^{-}$ intersects $c_{u}$ at $u=u_{j}^{-}+2u_{0}^{-}$”. Substituting $u=u_{j}^{-}+2u_{0}^{-}$ into the equality of $c^{-}$ and $c_{u}$, the result is obtained using the same calculation method as Formula (18):

(19) $$\begin{array}{l} \beta_{1}^{+}e^{\beta_{2}^{+}(u_{j}^{-}+2u_{0}^{-})}-\beta_{1}^{-}e^{\beta_{2}^{-}(u_{j}^{-}+2u_{0}^{-})}-\beta_{1}^{+}+\beta_{1}^{-}+2[\gamma_{1}^{+}\left(\frac{2}{1+e^{-\gamma_{2}^{+}(u_{j}^{-}+2u_{0}^{-}-\gamma_{3}^{+})}}-1\right)\\ -\gamma_{1}^{-}(\left(\frac{2}{1+e^{-\gamma_{2}^{-}(u_{j}^{-}+2u_{0}^{-}-\gamma_{3}^{-})}}-1\right))]+(k_{b}^{+}-k_{b}^{-})(u_{j}^{-}-2u_{0}^{-})+\frac{\delta_{k}^{-}}{\alpha^{-}}(1-e^{2\alpha^{-}u_{0}^{-}})\\ +f_{0}^{+}+f_{0}^{-}=0 \end{array}$$

The specific physical meanings of each parameter are listed in Table 3.

### 4.2. Parameter Identification of the Novel Algebraic Closure Model

#### 4.2.1. Hysteretic Model Parameter Identification Method

**Figure 10:**
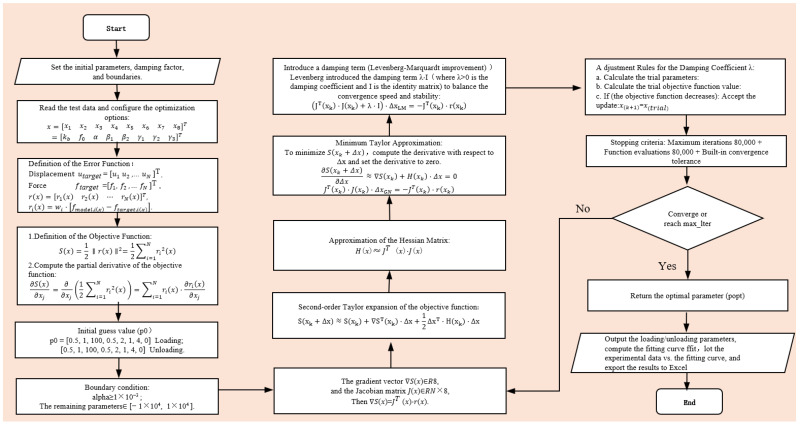
Flow chart of hysteretic model parameter identification.

The parameter identification of this hysteretic model adopts a function method based on solving the nonlinear least squares problem. Its core is to iteratively find the optimal solution using the Levenberg–Marquardt algorithm. The steps and procedure of the algorithm are shown in Figure 10:

#### 4.2.2. Result Comparison

**Figure 11:**
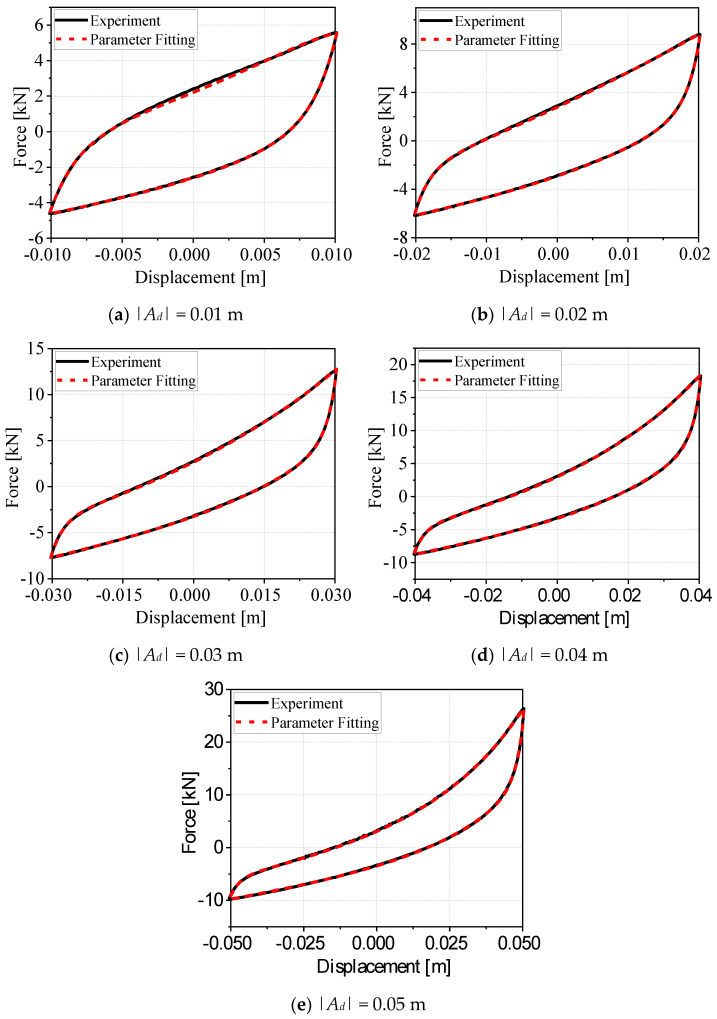
Comparison between experimental hysteresis curves and model-identified hysteresis curves of G300-type wire rope isolators (WRIs) at 0.2 Hz.

In this study, we performed parameter identification for the novel algebraic closed-form model. By comparing the experimental hysteresis curves at small and large displacement amplitudes with the model-predicted hysteresis curves obtained following parameter identification, we verified the calculation accuracy of the mechanical model calibrated with this set of identified parameters. Figure 11 shows the comparisons between the experimental and model-predicted curves of the G300 WRI under sinusoidal displacement excitation with no preload applied, in which the sinusoidal excitation was set to a frequency of 0.2 Hz, with displacement amplitudes of 0.01 m, 0.02 m, 0.03 m, 0.04 m, and 0.05 m, respectively. For each displacement amplitude, we obtained a two-stage (loading and unloading) dataset comprising eight parameters, with the corresponding data reported in Table 3. Here, + denotes the loading stage and—denotes the unloading stage. In the figure, black solid lines represent the experimental hysteresis curves, whereas red dashed lines represent the numerical simulation curves derived from the novel algebraic closed-form model through parameter identification. The good agreement between the two sets of curves confirms that this set of identified parameters can effectively reproduce the dynamic hysteretic mechanical behavior of the WRI, The parameter identification results are shown in Table 4.

**Figure 12:**
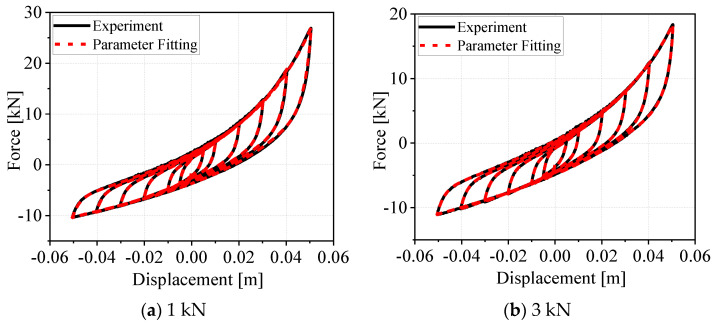
Comparison between experimental hysteresis curves and model-identified hysteresis curves of G300-type wire rope isolators (WRIs) under different pre-pressures.

Furthermore, to validate the applicability of the novel algebraic closed-form model, we analyzed the hysteresis curves of the G300 WRI under axial preloads of 1 kN and 3 kN. We separately identified the model parameters for the loading and unloading stages at different displacement amplitudes, with the corresponding data reported in Table 4, and subsequently performed a comparative analysis between the model-calculated results and experimental data. As shown in Figure 12, the experimental and model-predicted curves exhibit good agreement, which further validates the validity and robustness of both the novel algebraic closed-form model and the parameter identification method proposed in this study, The parameter identification results are shown in Table 5.

### 4.3. Constitutive Model of Fractional Derivative-Coupled Bouc-Wen Hysteresis

The proposed model integrates fractional dynamics (for memory effects) and nonlinear hysteresis (for loop shape), with the total restoring force equation given in Equation (20).

(20) $$F(t)=E_{0}x(t)+\eta \cdot D_{t}^{\alpha }x(t)+A\cdot z(t)+F_{0}$$

The definitions of each parameter are shown in Table 6.

#### 4.3.1. Fractional Derivatives for Fractal Memory Effects

To describe the memory effects implied by the hysteresis loop, we adopt the Grünwald–Letnikov fractional derivative (suitable for engineering applications),.defined in Equation (21).

(21) $$D_{t}^{\alpha }f(t)=\underset{h\to 0}{\lim}\frac{1}{h^{\alpha }}\sum_{k=0}^{\left[t/h\right]}{\left(-1\right)}^{k}\left(\begin{array}{c} \alpha \\ k \end{array}\right)f(t-kh)$$

where $\left(\begin{array}{c} \alpha \\ k \end{array}\right)=\frac{\Gamma (\alpha +1)}{\Gamma (k+1)\Gamma (\alpha -k+1)}$ (generalized binomial coefficient, calculated via the Gamma function to avoid cumulative errors).

This operator differs from integer-order derivatives: it depends on the entire historical loading process (not just the current state), perfectly matching the memory effects of fractal dynamic systems.

#### 4.3.2. Bouc–Wen Model for Nonlinear Hysteresis

The Bouc–Wen model is used to capture the strong nonlinearity of the hysteresis loop. Its internal state variable z(t) follows the differential equation as shown in Equation (22), The meanings of the parameters are shown in Table 7.

(22) $$\dot{z}(t)=\dot{x}(t)-\gamma |\dot{x}(t)|z|z{|}^{n-1}-\beta \dot{x}(t)|z{|}^{n}$$

**Figure 13:**
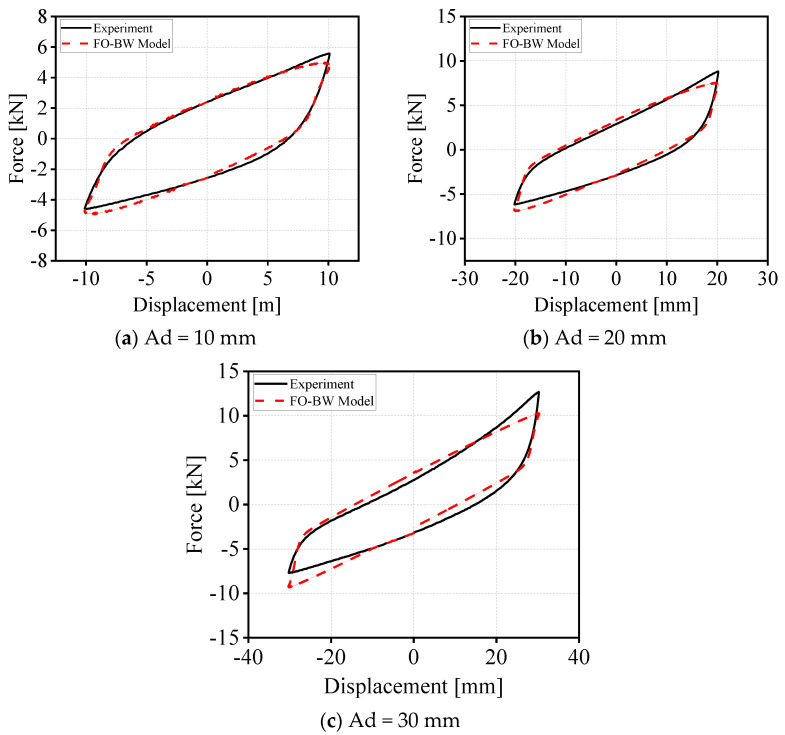
Comparison between experimental hysteresis curves and PO-WB model-identified hysteresis curves of G300-type wire rope isolators (WRIs) at 0.2 Hz.

This section focuses on the parameter identification of the established fractional-order coupled Bouc–Wen hysteretic model. To verify the reliability of the model calibration, the experimental hysteresis curves at different displacement amplitudes are compared with the model-predicted curves obtained after parameter identification. Figure 13 presents the comparison results for the G300 wire rope isolator (WRI) under 0.2 Hz sinusoidal displacement excitation without preload, with displacement amplitudes of 0.01 m, 0.02 m, and 0.03 m, respectively. The corresponding dataset is listed in Table 8. In the figures, the black solid lines represent the experimentally measured hysteresis curves, while the red dashed lines represent the model-fitted curves derived from the parameter identification process. The results show that the model-predicted curves are in high agreement with the experimental data under small displacement conditions. Even as the displacement amplitude increases, the two curves still maintain good consistency, with only minor deviations observed locally. Comprehensive analysis demonstrates that the model parameters obtained through parameter identification in this study can accurately and reasonably reproduce the dynamic hysteretic mechanical behavior of the wire rope isolator (WRI).

### 4.4. Comparison of Fitting Accuracy Between the Two Models 

To systematically compare and evaluate the fitting accuracy of the novel algebraic closed-form model and the fractional-order coupled Bouc–Wen hysteretic model, this study adopts the coefficient of determination (*R*^2^) and root-mean-square error (RMSE) as the core evaluation metrics. Quantitative assessments are conducted for typical operating conditions with displacement amplitudes of 10 mm, 20 mm, and 30 mm, respectively. By comparing the quantitative results listed in Table 9, it is evident that under various displacement amplitude conditions, the novel algebraic closed-form model maintains a consistently high R^2^ value, while its RMSE values are significantly lower compared to those of the fractional-order coupled Bouc–Wen model. This outcome thoroughly demonstrates that the novel algebraic closed-form model not only achieves a favorable reproduction of the experimentally measured hysteresis curves but also maintains excellent fitting stability and consistency during large-displacement and dynamic loading processes. Comprehensive analysis indicates that the proposed algebraic closed-form model exhibits superior fitting accuracy and robustness overall. It enables efficient and reliable characterization of the dynamic hysteretic mechanical behavior of wire rope isolators (WRI), thereby providing a solid theoretical foundation for subsequent parameter optimization and engineering applications.

## 5. Conclusions

This study systematically examined large-scale WRIs under varying displacement amplitudes, preload pressures, and excitation frequencies to explore how these factors influence their mechanical behavior. The experimental findings highlight significant variations in hysteretic characteristics, energy dissipation, the equivalent viscous damping coefficient, and average secant stiffness across different loading conditions. Moreover, the study identified performance differences between various WRI models. Additionally, the parameter identification for the novel algebraic closed-form hysteresis model demonstrated its accuracy and applicability under complex loading scenarios. The main conclusions are as follows:

Displacement Amplitude: The mechanical properties of wire rope isolators (WRIs) are significantly affected by the displacement amplitude. The dissipated energy *E_d_* increases with the displacement amplitude and has a positive correlation with structural parameter *A*. The viscous damping coefficient *v_e_* decreases monotonically as the displacement amplitude increases. The tensile stiffness varies depending on the model, while the compressive stiffness generally decreases with an increase in the displacement amplitude.

Preload Pressure: Preload pressure affects different WRI models differently. The *E_d_* values for all tested models increase with increasing displacement amplitude, but G280 is more sensitive to preload than G280S. The viscous damping coefficient *v_e_* decreases with higher displacement amplitudes, with more pronounced differences observed in the G280 model. The tensile stiffness responses differ across models, and while compressive stiffness decreases with displacement amplitude, G280S demonstrates greater stability across various preload conditions.

Excitation Frequency: Higher excitation frequencies enhance energy dissipation due to an increase in friction cycles and more pronounced plastic deformation. As a result, the *E_d_* value increases with frequency, while the *v_e_* value remains relatively unaffected by frequency changes. Tensile stiffness shows model-dependent variation, and compressive stiffness continues to decrease with increasing displacement amplitude, though the effect of frequency is minimal.

Algebraic Closed-Form Hysteresis Model: The results from the parameter identification of the algebraic closed-form hysteresis model accurately replicate the experimental hysteretic behavior, and the parameter identification process is deemed reliable.

In conclusion, this study provides a comprehensive understanding of how key loading parameters affect the mechanical properties of large-scale wire rope isolators, outlines the performance differences between various models, confirms the reliability of the novel closed-form model for simulation, and offers valuable theoretical and practical insights for the optimal and adaptive design of vibration isolation systems.

## Figures and Tables

**Table 1:** Geometric characteristics of WRIs.

WRIs	Length *L* (mm)	Height *H* (mm)	Width *W* (mm)	Diameter *D* (mm)	Parameter *A* (*H*/*W* × *D*)
G220	450	220	235	19.5	18.256
G240	450	240	260	19.5	18
G280S	500	280	290	21.5	20.759
G280	500	280	320	21.5	18.813
G300	500	300	320	21.5	20.156

**Table 2:** Axial testing conditions.

WRIs	NO. of Test	|*A_d_*| (mm)	*f* (Hz)	*P_N_* (kN)
G220	18	5, 10, 20, 30, 40, 50	0.2, 0.5, 1	0
	6	5, 10, 20, 30, 40, 50	0.2	1
	6	5, 10, 20, 30, 40, 50	0.2	3
G240	18	5, 10, 20, 30, 40, 50	0.2, 0.5, 1	0
	6	5, 10, 20, 30, 40, 50	0.2	1
	6	5, 10, 20, 30, 40, 50	0.2	3
G280S	18	5, 10, 20, 30, 40, 50	0.2, 0.5, 1	0
	6	5, 10, 20, 30, 40, 50	0.2	1
	6	5, 10, 20, 30, 40, 50	0.2	3
G280	18	5, 10, 20, 30, 40, 50	0.2, 0.5, 1	0
	6	5, 10, 20, 30, 40, 50	0.2	1
	6	5, 10, 20, 30, 40, 50	0.2	3
G300	18	5, 10, 20, 30, 40, 50	0.2, 0.5, 1	0
	6	5, 10, 20, 30, 40, 50	0.2	1
	6	5, 10, 20, 30, 40, 50	0.2	3

**Table 3:** Meaning of parameters.

Variable	Physical Meaning
$k_{e}^{\pm }$	Elastic tangent stiffness in the loading/unloading stage (including exponential and bell-shaped terms to simulate stiffness nonlinearity)
$k_{h}^{\pm }$	Hysteretic tangent stiffness in the loading/unloading stage (nonlinear attenuation, eventually converging to the constant $k_{b}^{\pm }$)
$u_{j}^{\pm }$	Internal variable in the loading/unloading stage (abscissa of the intersection point between $c^{\pm }$ and $c_{u}/c_{l}$)
$u_{0}^{\pm }$	Half-displacement difference in the loading/unloading stage (1/2 of the displacement difference between the start point and end point of $c^{\pm }$)
$f_{0}^{\pm }$	Intercept of the limit curve in the loading/unloading stage ($c_{u}$ intersection point of with the ordinate $f=f_{0}^{+}$, $c_{l}$ where is $f=-f_{0}^{-}$)
$\alpha^{\pm }$	Hysteretic stiffness attenuation rate parameter (α>0, the larger the value, the faster the attenuation)
$\beta_{1}^{\pm },\beta_{2}^{\pm }$	Elastic stiffness exponential term parameter (controlling the shape of the exponential curve)
$\gamma_{1}^{\pm },\gamma_{2}^{\pm },\gamma_{3}^{\pm }$	Elastic stiffness bell-shaped term parameter (controlling the amplitude, slope, and position of the bell-shaped curve)

**Table 4:** Parameters of the VRM used to reproduce hysteresis loops shown in Figure 11.

*A_d_* [mm]	Sgn ($\dot{d}$)	$k_{b}$	$f_{0}$	$f_{0}$ α	$\beta_{1}$	$\beta_{2}$	$\gamma_{1}$	$\gamma_{2}$	$\gamma_{3}$
10	+	3098.0456	33.44338	628.1991	130.4562	31.74804	21.31383	138.3865	0.013504
	−	141.9349	0.9709964	10,000	0.0074561	636.07266	2.1481754	164.74370	0.0145288
20	+	101.72148	1.9101646	464.83368	53.467992	7.0096209	1.1303681	0.4602343	1.7678767
	−	116.11579	28.448671	9999.9375	5.658 × 10^−5^	568.48764	16.268370	72.988602	0.0547046
30	+	49.399734	2.5688002	382.26648	10.373067	19.914453	0.0431679	17.283294	12.960494
	−	111.46909	37.507050	9999.7109	1.704 × 10^−6^	502.29980	21.050975	54.883879	0.0751294
40	+	105.06502	0.0474548	346.07251	5.0155421	29.085420	1.5215919	14.572084	20.286769
	−	106.10517	50.164864	9947.4423	1.203 × 10^−7^	448.19614	27.423659	49.191771	0.0886690
50	+	111.91218	0.2280102	319.06165	3.9428744	33.856765	1.4256575	35.701250	39.006317
	−	96.536814	59.737414	5790.5677	1.115 × 10^−7^	367.56694	32.440441	42.486367	0.1020543

**Table 5:** Parameters of the VRM used to reproduce hysteresis loops shown in Figure 12.

*P_N_* (kN)	*A_d_* [mm]	Sgn ($\dot{d}$)	$k_{b}$	$f_{0}$	$f_{0}$ α	$\beta_{1}$	$\beta_{2}$	$\gamma_{1}$	$\gamma_{2}$	$\gamma_{3}$
1 kN	10	+	114.469	7.63835	720.656	30.1153	11.2444	6.34953	16.23314	0.0683
		−	228.781	104.689	9999.97	2763.84	1.76 × 10^−5^	53.6494	603.849	0.01647
	20	+	88.3006	0.8494	523.981	0.7531	70.29445	12.6423	9.23683	0.00739
		−	164.888	24.2243	9999.72	1.4 × 10^−6^	744.6873	13.7500	110.796	0.04539
	30	+	168.213	2.8676	454.147	1.4903	52.7168	2.39399	7.878	3.433
		−	137.191	24.964	9856.28	2 × 10^−7^	573.337	14.507	67.586	0.0634
	40	+	144.863	0.0824	383.403	2.18146	44.129	1.1441	7.858	3.746
		−	113.419	37.624	10,000	1.3 × 10^−7^	449.236	21.273	48.093	0.0848
	50	+	139.792	0.889	362.131	2.177	43.65307	1.649586	32.55711	97.03974
		−	99.1033	52.24952	10,000	1.08 × 10	368.9830	28.91367	40.85283	0.1015
3kN	10	+	56.7003	7.3281	641.9842	25.2803	11.64494	3.242939	6.553435	1.695
		−	173.900	15.544	9998.764	2.75 × 10	1124.700	10.07429	205.1190	0.024
	20	+	126.145	1.888	493.8695	2.1899	36.84368	2.991173	1.017289	0.486
		−	156.893	15.453	9994.836	3.4 × 10^−7^	802.5710	10.08451	110.3041	0.0459
	30	+	37.308	9.4337	418.8315	1.284	46.9473	18.9211	5.733	0.087
		−	125.213	12.653	7841.204	1.46 × 10^−7^	574.0790	9.077324	52.31399	0.069631
	40	+	137.667	0.522	385.86	1.185	47.21297	0.325305	13.94472	25.31500
		−	103.170	15.4738	7078.010	1.05 × 10^−7^	446.239	10.909	39.349	0.0831
	50	+	127.926	0.02414	343.195	1.363	44.553	0.1726	7.4245	3.9785
		−	91.84048	18.003146	2588.8072	4.17 × 10^−7^	333.86376	12.366692	36.102	0.0899

**Table 6:** Detailed definition of each parameter.

Parameter	Physical Meaning	Unit (Example)
$F\left(t\right)$	Total restoring force of the wire rope isolator (output)	N (Newton)
$x\left(t\right)$	Displacement response of the isolator (input)	m (Meter)
$E_{0}$	Linear elastic stiffness, representing the inherent stiffness of the wire rope material	N/m
η	Fractional damping coefficient, quantifying the energy dissipation caused by fractal dynamics	N·s^α^/m
$D_{t}^{\alpha }$	Fractional differential operator of order α, core to describe long-range memory effects (a key feature of fractal dynamics)	-
α	Fractional order (0<α<1), characterizing the strength of memory effects: larger α means weaker memory, smaller α means stronger memory	-
A	Hysteresis force amplitude, controlling the maximum value of nonlinear hysteresis force	N
$z\left(t\right)$	Internal state variable of the Bouc-Wen model, recording the “historical loading information” to reflect hysteresis irreversibility	-
$F_{0}$	Zero-drift offset of the force sensor, correcting systematic errors in experimental measurements	N

**Table 7:** Additional parameter definitions for Bouc–Wen model.

Parameter	Physical Meaning
$\dot{z}\left(t\right)$	Time derivative of the internal state variable $z\left(t\right)$
$\dot{x}\left(t\right)$	Velocity of the isolator (time derivative of $x\left(t\right)$)
γ,β	Hysteresis shape parameters control the asymmetry and fullness of the hysteresis loop; larger values result in a more “square” loop.
n	Hysteresis smoothness index: controlling the sharpness of the loop’s vertex (larger n leads to sharper vertices, smaller n leads to smoother loops)

**Table 8:** Parameters of the FO-BW model used to reproduce hysteresis loops shown in Figure 13.

Displacement	*E*_0_	η	α	*A*	γ	β	*n*
10 mm	336.1622	471.3458	0.99	20,934.4527	0.1611	1.3911	1
20 mm	258.3322	222.8053	0.99	45,537.33	0.1666	1.0615	1
30 mm	240.856	85.9738	0.99	70,455.95	0.3504	1.6515	1.2189

**Table 9:** Comparison of fitting accuracy parameters between the new algebraic closure model and the fractional-order coupled Bouc–Wen model.

Displacement	Metric	PO-BW	VRM
10 mm	R^2^	0.9899	0.9926
	RMSE	0.3578	0.3059
20 mm	R^2^	0.9842	0.9968
	RMSE	0.6246	0.2881
30 mm	R^2^	0.9706	0.9981
	RMSE	1.1223	0.2859

## Data Availability

The original contributions presented in this study are included in the article. Further inquiries can be directed to the corresponding authors.

## References

[1] Liu Y., Shi D., Li Y., Liu S., He H., Chen H., Fan H. (2024). Ground Shock Attenuation Performances of Wire-Rope-Based Meta-Isolators. Thin-Walled Struct..

[2] Salvatore A., Carboni B., Chen L.-Q., Lacarbonara W. (2021). Nonlinear Dynamic Response of a Wire Rope Isolator: Experiment, Identification and Validation. Eng. Struct..

[3] Ledezma-Ramírez D.F., Tapia-González P.E., Brennan M.J., Paupitz Gonçalves P.J. (2022). An Experimental Investigation into the Shock Response of a Compact Wire Rope Isolator in Its Axial Direction. Eng. Struct..

[4] Rytömaa S., Malmi O., Laine S., Keinänen J., Viitala R. (2024). Wire Rope Isolator Identification and Dynamic Modeling for Small Amplitude Vibrations. Eng. Struct..

[5] Marin-Artieda C., Pardo-Ramos A. (2024). Energy Dissipation Enhancement of Wire Rope Isolators. Results Eng..

[6] Kang M.-S., Kim J.-H., Kim M.-H. (2023). Experimental and Numerical Study on the Vibration Characteristics of an Electric Switchboard with Wire Rope Isolators in Naval Ships. Ocean Eng..

[7] Tinker M.L., Cutchins M.A. (1992). Damping Phenomena in a Wire Rope Vibration Isolation System. J. Sound Vib..

[8] Demetriades G.F., Constantinou M.C., Reinhorn A.M. (1993). Study of Wire Rope Systems for Seismic Protection of Equipment in Buildings. Eng. Struct..

[9] Wang H.-X., Gong X.-S., Pan F., Dang X.-J. (2015). Experimental Investigations on the Dynamic Behaviour of O-Type Wire-Cable Vibration Isolators. Shock Vib..

[10] Balaji P.S., Moussa L., Rahman M.E., Vuia L.T. (2015). Experimental Investigation on the Hysteresis Behavior of the Wire Rope Isolators. J. Mech. Sci. Technol..

[11] He C., He K., Jiang L., Xie Q., Yang Z. (2024). Effects of Wire Rope Isolators on Seismic Life-Cycle Cost of UHV Bypass Switch. Int. J. Disaster Risk Reduct..

[12] Barbieri N., Barbieri R., Da Silva R.A., Mannala M.J., Barbieri L.D.S.V. (2016). Nonlinear Dynamic Analysis of Wire-Rope Isolator and Stockbridge Damper. Nonlinear Dyn..

[13] Leblouba M., Rahman M.E., Barakat S. (2019). Behavior of Polycal Wire Rope Isolators Subjected to Large Lateral Deformations. Eng. Struct..

[14] Wang Z.-J., Zang J., Zhang Z., Song X.-Y., Zhang Y.-W., Chen L.-Q. (2024). Nonlinear Broadband Vibration Reduction of Nitinol-Steel Wire Rope: Mechanical Parameters Determination and Theoretical-Experimental Validation. Mech. Syst. Signal Process..

[15] Yang S., Fan W., Liu Y., Ma L., Chen Z. (2025). A High-Static-Low-Dynamic Stiffness Wire Rope Isolator with a Magnetic Negative Stiffness Device: Experiment, Analytical Method, and Optimization. Eng. Struct..

[16] Vaiana N., Sessa S., Paradiso M., Marmo F., Rosati L., Carcaterra A., Paolone A., Graziani G. (2020). An Efficient Computational Strategy for Nonlinear Time History Analysis of Seismically Base-Isolated Structures. Proceedings of the XXIV AIMETA Conference 2019, Rome, Italy, 15–19 September 2019.

[17] Brewick P.T., Farzad R. (2025). Hierarchical Bayesian Calibration of Bouc–Wen Hysteretic Models with Applications to Seismic Isolators. Mech. Syst. Signal Process..

[18] Zhang Y., Lei Y., Cao J., Liu Q., Liao W.-H. (2025). Nonlinear Wire Rope Isolator with Magnetic Negative Stiffness. Mech. Syst. Signal Process..

[19] Orgéas L., Favier D. (2001). Stress State Effect on Mechanical Behaviour of Shape Memory Alloys: Experimental Characterisation and Modelling. J. Phys. IV France.

[20] Bles G., Nowacki W.K., Tourabi A. (2009). Experimental Study of the Cyclic Visco-Elasto-Plastic Behaviour of a Polyamide Fibre Strap. Int. J. Solids Struct..

[21] Vandenbroucke A., Laurent H., Aït Hocine N., Rio G. (2010). A Hyperelasto-Visco-Hysteresis Model for an Elastomeric Behaviour: Experimental and Numerical Investigations. Comput. Mater. Sci..

[22] Wen Y.-K. (1976). Method for Random Vibration of Hysteretic Systems. J. Eng. Mech. Div..

[23] Wen Y.K. (1980). Equivalent Linearization for Hysteretic Systems Under Random Excitation. J. Appl. Mech..

[24] Song J., Der Kiureghian A. (2006). Generalized Bouc–Wen Model for Highly Asymmetric Hysteresis. J. Eng. Mech..

[25] Karabutov N., Shmyrin A. (2020). Parameters Adaptive Identification of Bouc-Wen Hysteresis. IFAC-PapersOnLine.

[26] Sireteanu T., Giuclea M., Mitu A.-M., Ghita G. (2012). A Genetic Algorithms Method for Fitting the Generalized Bouc-Wen Model to Experimental Asymmetric Hysteretic Loops. J. Vib. Acoust..

[27] Vaiana N., Spizzuoco M., Serino G. (2017). Wire Rope Isolators for Seismically Base-Isolated Lightweight Structures: Experimental Characterization and Mathematical Modeling. Eng. Struct..

[28] Vaiana N., Rosati L. (2023). Classification and Unified Phenomenological Modeling of Complex Uniaxial Rate-Independent Hysteretic Responses. Mech. Syst. Signal Process..

[29] Vaiana N., Sessa S., Marmo F., Rosati L. (2018). A Class of Uniaxial Phenomenological Models for Simulating Hysteretic Phenomena in Rate-Independent Mechanical Systems and Materials. Nonlinear Dyn..

[30] Kumar S.S., Krishna A.M., Dey A. (2017). Evaluation of Dynamic Properties of Sandy Soil at High Cyclic Strains. Soil Dyn. Earthq. Eng..

[31] Pellecchia D., Vaiana N., Spizzuoco M., Serino G., Rosati L. (2023). Axial Hysteretic Behaviour of Wire Rope Isolators: Experiments and Modelling. Mater. Des..

